# The Diagnostic and Prognostic Role of Interleukin 6 and Interleukin 8 in Childhood Acute Gastroenteritis—A Review of the Literature

**DOI:** 10.3390/ijms25147655

**Published:** 2024-07-12

**Authors:** Heidrun Adumitrăchioaiei, Maria Oana Săsăran, Cristina Oana Mărginean

**Affiliations:** 1 Department of Pediatrics I, University of Medicine, Pharmacy, Sciences and Technology George Emil Palade from Târgu Mureș, Gheorghe Marinescu Street No. 38, 540136 Targu Mures, Romania; ad.heidi91@gmail.com (H.A.); marginean.oana@gmail.com (C.O.M.); 2 Department of Pediatrics III, University of Medicine, Pharmacy, Sciences and Technology George Emil Palade from Târgu Mureș, Gheorghe Marinescu Street No. 38, 540136 Targu Mures, Romania

**Keywords:** acute gastroenteritis, interleukin 6, interleukin 8, biomarker, diagnosis, severity

## Abstract

Acute gastroenteritis in pediatric patients represents a major cause of morbidity and mortality in children. Interleukins 6 (IL-6) and 8 (IL-8) have been intensely studied in relation to various inflammatory conditions, including acute gastroenteritis, as they are activated in response to infection. This review aims to evaluate the ability of IL-6 and IL-8 to distinguish between bacterial and viral etiologies of acute gastroenteritis in children and to assess whether their levels correlate with the severity of this condition in light of currently available data. A scientific database search was performed to identify studies that investigated the role of IL-6 and IL-8 in acute gastroenteritis in the pediatric population. We identified nine studies that matched the review’s objective. Both cytokines show increased values in acute gastroenteritis, but IL-6 levels are significantly higher in cases of bacterial infections. IL-8 levels do not present an increase to the same extent in cases of bacterial diarrhea in children but seem to be associated with the severity of the disease. The lack of sufficient research focusing on IL-6 and -8 as diagnostic, prognostic and severity biomarkers of acute gastroenteritis in children leaves room for further research on this topic, which must include larger cohort studies.

## 1. Introduction

Acute gastroenteritis is a diarrheal disease with rapid onset, the etiology of which is dominated by viral infections, regardless of the level of socio-economic development. Gastroenteritis is associated with increased rates of mortality and morbidity due to related complications, mainly represented by severe dehydration [1,2,3]. Malnutrition associated with acute gastroenteritis is an additional factor that contributes to morbidity and mortality among pediatric patients [4,5,6]. Still, the malnutrition–gastroenteritis relationship has negative effects from both sides. In a study conducted on a group of 335 children under the age of 6 hospitalized for moderate or severe malnutrition, almost 70% suffered acute diarrheal disease as a complication at hospitalization [4,7]. As a matter of fact, gastroenteritis has been held accountable for approximately 10% of pediatric deaths worldwide, ranking second among causes of death in children [8]. In terms of transmission routes, fecal–oral contact, person-to-person contact and contaminated food/water ingestion have been described [9]. Depending on its duration, diarrhea can be classified into acute diarrhea and persistent diarrhea [1,10,11].

Etiologies of gastroenteritis include both viral and bacterial infections, with viral infections being accountable for most cases of acute diarrhea, especially at young ages. Rotavirus, along with norovirus, is accountable in the US for 58% of cases of acute viral gastroenteritis, followed by adenovirus, sapovirus and astrovirus [12,13]. The main cause of diarrhea was rotavirus infection until the appearance of a vaccine. Rotavirus infection registered a significant increase from 1993 (20%) until 2015, when it was found in 64% of all cases of acute gastroenteritis. Currently, in areas where rotavirus vaccination is widely performed, norovirus has become the main causative agent of acute diarrheal disease [14,15,16].

Bacterial etiologies are different in developed versus developing countries. Thus, *enterotoxigenic E. coli* (*ETEC*) is the most important bacterial agent identified in children with acute gastroenteritis in developing areas, followed by *Campylobacter*, *Salmonella* and *Shigella*. In developed countries, *Campylobacter*, *Salmonella*, *Shigella* and *enterohemorrhagic Escherichia coli* (*EHEC*) are the main bacterial agents incriminated in the etiology of acute gastroenteritis, representing 10–20% of all diarrhea cases [13].

Stool culture, the current gold-standard investigation when bacterial infection is suspected, requires an interval of 1–2 days for completion, during which time the absence of an etiological diagnosis increases the risk of morbidity and mortality. Thus, empirical antibiotic therapy is often started. At a time when antibiotic resistance is a global health problem, empirical antibiotic use in acute gastroenteritis is worrying—a fact that confirms the need for a rapid etiological diagnosis of this pathology, which can be achieved by monitoring levels of interleukin (IL)-6 [17]. The development of a rapid and inexpensive protocol in acute diarrheal disease would help to direct a targeted therapy and reduce the misuse of antibacterial medication [3,18].

Cytokines are directly involved in the pathogenesis of viral infections and acute gastroenteritis. It is well known that intestinal epithelial cells secrete numerous cytokines in the presence of stimuli of bacterial or viral origin and in local, chronic inflammation [19,20]. In particular, different studies have tried to establish a correlation between IL-6 and IL-8 levels, the acute phase of gastroenteritis, and the ability to distinguish its etiologies [21]. The ratio between IL-8 and C-X-C motif chemokine ligand 8 (CXCL 8) [IL-8/CXCL 8] increases in the acute phase of inflammation caused by bacteria, viruses or parasites in the gastrointestinal tract, which is why it has been proposed as a diagnostic or treatment biomarker in multiple inflammatory pathologies of the digestive system [22]. There has been particular interest in the role of IL-6 and IL-8 in intestinal inflammation, with inflammatory bowel disease standing as a classical pathology in which the two cytokines play a significant role. In Crohn’s disease, IL-6 levels have been associated with the severity, frequency of relapses and clinical activity of the disease, whereas IL-8 has been associated with the inflammation degree of the colon mucosa [23,24]. The possibility of CXCL 8 contributing to the body’s defense mechanisms against bacterial infections has been discussed. Studies of IL-8 genotypes could facilitate the development of targeted therapeutic regimens and possibly vaccines and thus reduce mortality caused by acute diarrheal diseases [25]. IL-6, an essential component of the acute-phase reaction, shows high values in bacterial infections [26], with rapid increases during sepsis, and its levels correlate with the degree of diarrhea severity. IL-6 might also help in the differentiation of bacterial from non-bacterial etiologies in acute gastroenteritis [26]. Expert studies report elevated levels of IL-6 and IL-8 in gastroenteritis caused by rotavirus, which reinforces the idea that measurement of these interleukins could be a real tool in treating children with acute gastroenteritis [27,28,29,30]. However, research on IL-6 and IL-8 as biomarkers in acute diarrheal disease is limited.

This review aims to investigate the role of IL-6 and IL-8 in correlating the clinical picture with inflammatory status in acute gastroenteritis in the pediatric population and in distinguishing its etiologies in light of recent data.

## 2. Methods

The PubMed, Google Scholar and Web of Science (WOS) databases were searched for articles published up to 1 December 2023 that analyzed a relationship between acute gastroenteritis in the pediatric population and IL-6 and IL-8. The search terms were as follows: “gastroenteritis in children” OR “acute gastroenteritis in children” OR “diarrhea” AND “interleukin 6” OR “interleukin” OR “interleukin 8”.

The inclusion criteria were population-based human studies, English-language-based studies and research articles that analyzed the correlation between serum levels of IL-6 and IL-8 and acute gastroenteritis in children.

Full-length papers consisting of randomized controlled trials (RCTs), prospective cohort studies, retrospective cross-sectional studies and longitudinal studies were included.

We excluded studies that did not meet the objective of our article, case reports, editorials, review articles and meta-analyses, abstracts, articles without freely available abstracts, and duplicates/triplicates. Furthermore, we excluded articles and accompanying abstracts written in a language other than English, adult-based population studies and studies that focused on cytokine levels in childhood acute gastroenteritis without including IL-6 or IL-8.

**Figure 1:**
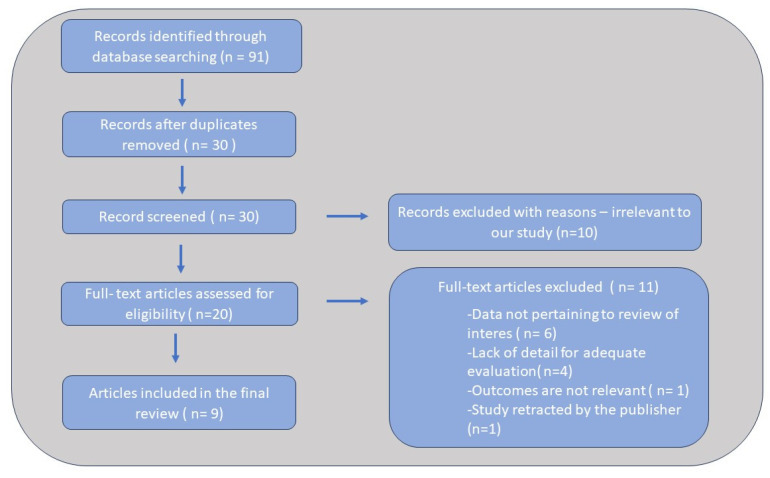
Flowchart of the article selection process.

The article selection process initially consisted of the removal of duplicates and triplicates from the three databases. Afterwards, HA and MOS analyzed each abstract in order to assess the type of the article and its compatibility with the review’s objectives. Case reports, reviews, meta-analyses and abstracts were excluded, as well as irrelevant articles. Afterwards, the three authors analyzed the complete articles and selected the studies which met the inclusion and exclusion criteria. Eventual disagreements between the three authors were solved, and each of the three authors agreed to the inclusion of each individual record. A flowchart with a schematic representation of the article selection process has been provided in Figure 1. For each article that was part of the final selection, we recorded the following information: author name and year of publication, type of study, study population and study group division, and main findings in terms of target cytokine levels.

## 3. Results

We identified 10 studies that examined the association between levels of IL-6 and IL-8 and acute gastroenteritis in children: 4 studies included only IL-6 levels, 1 study analyzed IL-8 and 4 studies investigated the relationship between serum levels of both IL-6 and IL-8 and acute gastroenteritis in children. One of the studies which was retracted by the publisher was left out of the final selection pool, leaving nine studies in the end.

### 3.1. Interleukin 6: Structure and Function 

IL-6 is a 21–28 kDa glycosylated protein with three receptor binding sites and a four-helix structure, and its gene is located on chromosome 7p21. The receptor complex is composed of a type I transmembrane glycoprotein, which is the IL-6 receptor-binding chain, and the transmembrane signal transducer protein type GP130 [31,32,33]. As a common receptor component of the IL-6 cytokine family, Gp130 is found in the liver, lungs, spleen and heart [34]. The IL-6 family comprises 10 ligands and 9 receptors, and these have a common core structure, which is responsible for a mutual signal transducer in their receptor complex, and multiple roles [34]. The Il-6–IL-6R axis seems to be responsible for inflammatory process maintenance and the progression of inflammatory diseases, such as rheumatoid arthritis [35].

The involvement of IL-6 in the maturation of naive B lymphocytes into plasma cells and in the differentiation of cytotoxic T lymphocytes suggests that IL-6 promotes pathological inflammatory responses and systemic autoimmunity [36]. Currently, the role of IL-6 as a pro-inflammatory cytokine, which contributes to the activation of innate and adaptive immune responses, is widely accepted. Stromal cells and leukocytes release IL-6 as part of the innate immune response; IL-6 is thus responsible for modulating the immune response of B and T lymphocytes. At the same time, IL-6 is also produced by non-lymphoid cells, which explains the numerous anti-inflammatory and regenerative functions that have also been attributed to it [37,38,39,40,41]. IL-6 is documented in various inflammatory pathologies, starting with acute gastroenteritis, autoimmune diseases, systemic juvenile idiopathic arthritis, rheumatoid arthritis, inflammatory bowel diseases and colorectal cancer [33,34,36,42]. In studies performed on acute infections, the capacity of IL-6 to facilitate antimicrobial defense and to limit excessive tissue damage has been shown, and IL-6 is responsible for maintaining chronic inflammation [43,44,45,46]. IL-6 is also involved in maintaining the integrity of tissues and organs, including the integrity of the intestinal epithelial barrier [44,47,48]. Impairment of the integrity of this barrier has been shown to be related to disruption or blockade of IL-6R signaling [49,50,51].

### 3.2. Interleukin 8: Structure and Function

IL-8 was first studied about 30 years ago, when three different teams of researchers described at about the same time what is now called IL-8 or CXCL8, a low-molecular-weight protein [22]. Two N-terminal cysteine residues separated by a single amino acid in the primary amino acid sequence of IL-8 lead to the appearance of two disulfide bonds in the higher-level amino acid structure, and thus it has been identified as belonging to the CXCL chemokine family [22,52,53]. IL-8 binds with high affinity to the G protein-coupled receptors CXCR1 and CXCR2, and the Duffy antigen receptor for cytokines, CXCR1, formerly known as the IL-8 type A receptor, which responds to high concentrations of IL-8, is most often found at sites of infection. The former type B IL-8 receptor, now known as CSCR2, initiates neutrophil migration away from the site of inflammation [54,55]. The secretion of IL-8 therefore causes the activation of peripheral blood neutrophils, which will migrate to a site of infection, facilitating the elimination of pathogens. IL-8 is considered a vital mediator in acute neutrophil-mediated inflammation, both in vivo and in vitro [54,56]. An increased secretion of IL-8 may be responsible for the appearance of augmented inflammation in patients with chronic inflammatory pathologies. Conversely, blocking IL-8 secretion contributes to chronic inflammation through a delayed neutrophil response [57,58,59].

CXCL 8 has an important role in the body’s antimicrobial defense mechanisms. Studies show the presence of IL-8 in acute and chronic inflammatory conditions occurring in the gastrointestinal tract. CXCL 8 is expressed on epithelial cells, fibroblasts, macrophages, monocytes, neutrophils and endothelial cells. The role of CXCL 8 in gastrointestinal pathophysiology is incompletely understood, as IL-8 expression has been found in both invading and resident cells of the gastrointestinal tract (neutrophils, macrophages, monocytes, fibroblasts, endothelial cells and epithelial cells). IL-8 values have been proposed as diagnostic and/or prognostic markers in several diseases of the gastrointestinal tract—not only acute but also chronic pathologies, such as gastric and colonic carcinomas and inflammatory bowel diseases—due to its promotion of inflammation and neoplasia [22]. 

### 3.3. Interleukin 6 and Interleukin 8: Diagnostic Role in Childhood Acute Gastroenteritis

An obvious involvement of IL-6 in intestinal inflammation has been proven in animal models. Furthermore, target modulation of various cytokines, including IL-6, has been proposed as a therapeutic option in inflammatory bowel disease [60]. IL-8 is another cytokine activated in response to infection which favors the chemotaxis of inflammatory cells [61]. Furthermore, IL-8 seems to play an important role in the development of intestinal inflammation, including inflammatory bowel disease, as well as in the development of intestinal malignancies [22]. Thus, taking into consideration the conjoint role of Il-6 and IL-8 as part of intestinal inflammation and the cytokine cascade of acute infections, various studies have focused on the diagnostic biomarker role of the two cytokines in childhood acute gastroenteritis. These studies are summarized in Table 1.

A case–control study revealed that serum levels of both IL-6 and IL-8 were significantly higher in children with acute gastroenteritis compared to the control group (Table 1). According to ROC analysis, serum IL-6, in contrast to serum IL-8, presented a high discriminatory power for distinguishing between bacterial and viral etiologies. Furthermore, stool levels of both IL-6 and IL-8 were higher in gastroenteritis patients when compared with healthy controls, but due to technical difficulties linked to the enzyme-linked immunosorbent assay detection method, the biomarker characteristics of these fecal cytokines are debatable [37]. Another study highlighted the strong sensitivity and specificity performance of serum IL-6 in differentiating between viral and bacterial gastroenteritis, in contrast to IL-8, which presented similar increases in serum values of patients with gastroenteritis, regardless of etiology [19]. The same two cytokines were the focus of another, similar study which identified a correlation between serum IL-6 and disease severity, fecal IL-6 and maximum number of daily bowel movements, as well as serum IL-8 and fever duration. Significantly higher serum levels of IL-8 were found in subjects with rotavirus acute diarrhea when compared with those infected with norovirus. However, as in other studies, IL-6 was the only one of the two cytokines that was able to aid in the differential diagnosis between bacterial and viral acute diarrhea [20]. Vaisman et al. once again confirmed these findings, proving that both serum and fecal IL-6 levels increased to a higher extent in bacterial gastroenteritis, and they also found a correlation between serum IL-6 and the positivity rate of bacterial culture. Nevertheless, serum IL-8 levels also increased in patients with acute gastroenteritis and presented a tendency towards more significant augmentation in those patients with bacterial infections [62]. In an analysis of a group of 147 children with acute diarrheal disease, it was demonstrated that IL-6, IL-8 and C reactive protein (CRP) had significantly higher values in patients with bacterial enterocolitis, and, notably, IL-6 showed higher sensitivity, specificity and positive predictive value than the other two inflammatory markers [63].

Given the proven role of IL-6 in the modulation of the immune response triggered by various infectious agents and in the maintenance of the structural integrity of the epithelial intestinal barrier, studies analyzing variations in IL-6 in childhood acute gastroenteritis have surfaced recently. The analysis of IL-6 from blood samples based on a cross-sectional study proved that this cytokine showed significantly higher values in the group diagnosed with diarrhea of bacterial origin compared to the group in which the etiology of diarrhea was non-bacterial (*p* < 0.05). It should be noted that the percentages of those with bacterial and viral diarrhea were approximately equal, and of the 48.8% of the total subjects diagnosed with bacterial etiological agents, *Escherichia coli* was identified in 33% of cases and *Salmonella* in 20.5% of patients [25].

Xu et al. included in their study 120 patients aged between 2 months and 7 years who presented with symptoms of acute diarrheal disease. Following the controversial results of the study, they concluded that levels of IL-6 were lower in patients with bacterial etiological agents compared to viral ones [64]. Moreover, Mangiarotti et al. concluded that IL-6 concentration cannot be used to differentiate rotavirus from bacterial gastroenteritis within a childhood population [65]. This finding contrasted with other data in the literature which showed significantly greater increases in IL-6 levels in cases of bacterial etiology [19]. Furthermore, one study conducted on children with acute diarrheal disease caused by rotavirus brought to light that most of the patients (more than 70%) presented no positivity of serum IL-6 [66]. Thus, this evidence shows a lack of significant inflammatory status in viral gastroenteritis characterized by a lack of significant increase in inflammatory cytokines. So far, only one study has considered the assessment of IL-8, without concomitantly taking into consideration the role of IL-6 as well. Azim et al. showed in a study that IL-8 cannot be used in the differential diagnosis of gastroenteritis produced by rotavirus and gastroenteritis without an identified etiological agent because it did not register significant increases in the group of patients with rotavirus infection [28].

**Table 1:** Summary of studies analyzing IL-6 values in relation to bacterial and viral gastroenteritis.

No.	Reference/Author/Year	Type of Study	Population and Age Group Assignment	Conclusions
1.	Zaki et al., 2020 [37]	Case–control	150 patients, aged under 10, divided as follows: 50 patients with acute bacterial gastroenteritis 50 patients with acute viral gastroenteritis 50 healthy individuals—the control group	- IL-6 a possible marker for acute gastroenteritis of bacterial etiology with high discriminatory power for distinguishing between bacterial and viral etiologies, according to ROC analysis - Both IL-6 and IL-8 showed significantly higher serum values in the group of patients with acute gastroenteritis versus the control group (*p* < 0.001) - Stool levels of both IL-6 and IL-8 were increased in both groups with acute gastroenteritis, but due to technical limitations it is unclear whether these can be used as differentiating biomarkers between bacterial and viral gastroenteritis
2.	Herlina et al., 2016 [25]	Cross-sectional	80 children with acute diarrhea, aged between 1 and 5 years, divided as follows: 41 boys 39 girls	- Serum IL-6 showed significantly higher values in the group of those with acute gastroenteritis of bacterial origin versus the group with non-bacterial etiology
3.	Xu et al., 2018 [64]	Case–control	120 patients with acute gastroenteritis, divided as follows: 50 patients with acute bacterial gastroenteritis 70 patients with acute viral gastroenteritis	- PBMC IL-6 mRNA transcription, IL-6 serum levels and IL-6 protein expression were higher in the group of patients with acute viral versus bacterial gastroenteritis (*p* < 0.001)
4.	Lin et al., 2006 [19]	Case–control	56 patients, divided as follows: 18 patients with acute bacterial gastroenteritis 21 patients with acute viral gastroenteritis 17 healthy, married individuals	- Serum IL-6 showed significantly higher values in the group of patients with acute bacterial gastroenteritis versus the group of those with acute viral gastroenteritis (*p* <0.001) - Serum Il-8 increased in both bacterial and viral gastroenteritis without presenting important variations in relation to gastroenteritis etiology - Unlike IL-8, IL-6 presented good sensitivity and specificity parameters
5.	Chen et al., 2014 [20]	Case–control	99 patients, aged between 4 months and 14 years, divided as follows: 66 patients with acute viral gastroenteritis 23 patients with acute bacterial gastroenteritis 10 healthy individuals—the control group	- Serum IL-6 moderately correlated with disease severity - Correlation between fecal level of IL-6 and maximum number of daily bowel movements - Serum Il-6 capable of differentiating bacterial from viral gastroenteritis - Serum IL-8 level correlated with fever duration - IL-8 showed higher values in patients diagnosed with rotavirus compared to those with norovirus
6.	Vaisman et al., 2003 [62]	Case–control	33 patients, aged between 6 months and 6 years, with acute invasive gastroenteritis and 7 healthy, married individuals, divided as follows: 22 patients with proven bacterial etiology 11 patients with unproven bacterial etiology 7 healthy individuals—the control group	- IL-6 showed higher serum values in patients with gastroenteritis versus the control group (*p* < 0.001) - IL-6 showed higher serum values in patients with bacterial gastroenteritis versus the control group - Stool IL-6 in the group with proven bacterial etiology was significantly higher (*p* < 0.05) - IL-8 showed higher serum values in patients with gastroenteritis versus the control group - IL-8 tended to present higher values in the group of those with bacterial versus viral etiology
7.	Yeung et al., 2004 [63]	Case–control	147 patients with acute gastroenteritis, divided as follows: 115 patients with an identified agent, 72 with bacterial etiology and 43 with viral etiology 32 patients without an identified agent	- IL-6, IL-8 and CRP were significantly higher in bacterial enterocolitis subjects - IL-6 presented the best sensitivity, specificity and positive predictive value for bacterial etiologies
8.	Azim et al., 2002 [28]	Case–control	40 patients aged between 7 months and 24 months with acute gastroenteritis, divided as follows: 29 patients with acute rotavirus gastroenteritis 11 patients with gastro-enteritis without identified etiological agents	- IL-8 did not show significant increases in the group of those with rotavirus compared to the group without an identified etiology
9.	Mangiarotti et al., 1999 [65]	Cohort study	119 children hospitalized for diarrhea: 60 patients with rotavirus diarrhea 59 patients with bacterial diarrhea	- IL-6 failed to differentiate viral from bacterial diarrhea

**Legend**: CRP—C reactive protein; IL-6—interleukin 6; mRNA—messenger RNA; PBMCs—protein expression in peripheral blood mononuclear cells; ROC—receiver operating characteristic.

## 4. Discussion

In the presence of inflammation—in our case, the inflammation of the gastrointestinal tract caused by acute gastroenteritis—a release of IL-6 takes place to activate the body’s defense system [67]. IL-6 is also an essential cytokine for maintaining the integrity of the mucosal barrier and for the proliferation of epithelial cells, playing a role in the immune response which takes place in the intestinal microenvironment [68,69]. Intraepithelial lymphocytes stimulated by the intestinal microbiome stimulate the release of IL-6 to preserve the integrity of the mucosa. The absence of IL-6 in mice resulted in thinning of the intestinal mucosal layers, along with an increase in intestinal paracellular permeability [69]. In the context of intestinal inflammation, the integrity of the intestinal epithelial barrier is affected by increasing intestinal permeability [70]. IL-8 also seems to play a very important role in the intestinal inflammation caused by enteric infections. Casola et al. have showed that the rotavirus-triggered activation of protein-(AP) 1 and nuclear factor-(NF) kappaB elements are mandatory for the activation of the IL-8 promoter [71]. Moreover, one study showed that both enteroaggregative and enterotoxigenic *E. coli* can induce increased expression and fecal elimination of IL-8, highlighting once again this cytokine’s involvement in the inflammation cascade triggered by enteric pathogens [72]. IL-8 mRNA expression has also been shown to be reduced in patients with *Salmonella* gastroenteritis under statin administration. Thus, a statin concentration between 10 and 20 µM led to a reduction in IL-8 mRNA expression, while values above 50 µM were associated with improvement of IL-8 mRNA expression [73]. Thus, statin-driven reduction in inflammatory status also produced a downregulation of IL-8 mRNA expression.

The release of IL-6 and -8 in a case–control study that included three study groups (a control group of healthy children, well-nourished children with acute gastroenteritis and malnourished children with acute diarrhea) was more reduced in the group of malnourished children with gastroenteritis compared to the group of those with normal weight and acute gastroenteritis [74]. This study brings to light a weaker or absent inflammatory response of the malnourished body during episodes of gastroenteritis.

As exposed in the results of this review, most of the studies available so far have focused on the ability of IL-6 and IL-8 to differentiate viral and bacterial etiologies of acute diarrhea in children, as well as their correlation with symptom duration and severity [28,62,63]. Antibiotic resistance is a problem among low- and middle-income countries, due to a wide range of factors, such as the uncontrolled use of these drugs, limited access to medical personnel and specialist investigations, poor hygiene, and the erroneous coordination of waste and waste emissions from antibiotic production [75,76]. As a matter of fact, a study carried out on 88 patients with acute gastroenteritis (25% of the subjects with bacterial etiology and 75% with viral etiology) even supported the use of empirical antibiotic therapy [17]. However, a study carried out in Kenya over a period of 7 years, whose target population group was infants and children aged between 2 and 59 months, observed that approximately 30% of all patients received unjustified antibiotic therapy. Insufficient antibiotic prescription duration was recorded in a significant percentage of patients—a fact that supports the need to cease the unjustified or incorrect administration of antibiotics as soon as possible among children with diarrhea [77], at a time when antibiotic resistance has become a real health problem worldwide [75,76]. Thus, a more rapidly achievable diagnosis could avoid unnecessary antibiotic administration, and accurate representation of inflammation through cytokine levels, quantifiable from sera, could aid in therapeutic management.

IL-6 increase has also been related to complications of acute gastroenteritis. In a 1-year study in Mongolia, IL-6 and TNF-alpha levels were studied in infants with benign seizures and acute gastroenteritis and compared with those without seizures and gastroenteritis. The results showed significantly higher serum levels in the group of those with seizures compared to those without these neurological manifestations, and IL-6 and TNF-alpha values were correlated with the severity of seizures [78]. Consequently, this study showed that one of the frequent possible complications of acute gastroenteritis in young children, the febrile seizure, can determine a supplemental increase in cytokine level. IL-6 concentration was also determined during an epidemic caused by Shigella dysenteriae 1, along with TNF. IL-6 concentrations were significantly higher in children who developed complications following infection with Shigella dysenteriae 1 compared to the group of children infected with the same bacterium but without complications in the first and second weeks after infection [79]. An increase in liver enzymes has been observed in children with gastroenteritis caused by rotavirus—an increase that can in some cases lead to the appearance of hepatitis, most often in a mild form [80,81]. Starting from the premise that IL-6 is associated with increased values in acute gastroenteritis regardless of the etiological nature, a study analyzed liver enzyme values and IL-6 values in cases of rotavirus infection with the aim of identifying a possible association between liver enzyme levels and IL-6 values. The analysis of liver enzymes in children with acute gastroenteritis in parallel with levels of IL-6 led to the conclusion that increased alanine aminotransferase (ALT) and aspartate aminotransferase (AST) values in the context of acute gastroenteritis can be correlated with increased IL-6. Thus, in cases when IL-6 cannot be determined, liver enzyme levels can give a hint towards IL-6 values [82]. This study once again confirms the importance of IL-6 in evaluating the degree of severity of acute gastroenteritis in real time. Thus, these data suggest that a supplementary augmentation of IL-6 release accompanies complications of acute gastroenteritis in children.

The main limitation of our review is the small number of studies included due to the paucity of research in the available literature. This pitfall, which impairs accurate assessment of the utility of cytokines in the differential, etiology-based diagnosis of gastroenteritis, was also acknowledged by the most recent European Society of Pediatric Gastroenterology, Hepatology and Nutrition (ESPGHAN) guidelines. According to this societal paper, IL-6 and IL-8 cannot be used for the differential diagnosis of acute diarrhea in children, and C reactive protein (CRP), although less reliable than procalcitonin, apparently performed better in distinguishing viral from bacterial etiologies [83]. Still, other infant-based research has showed how CRP is more trustable in systemic bacterial infections [84]. The promising results of some studies cannot be neglected though. As several studies have shown, significantly higher values of cytokine levels have been reported in the case of bacterial infections. In particular, IL-6 has been distinguished as a fast, cheap and safe marker in the etiological diagnosis of acute gastroenteritis and can thus help to prevent the use of unfounded antibacterial therapy in the treatment of this pathology and facilitate treatment decisions [17,37,62]. Therefore, future studies are warranted to clarify further the diagnostic and prognostic role of cytokines in childhood acute gastroenteritis.

## 5. Conclusions

Defined as a global health problem, acute gastroenteritis requires the implementation of rapid diagnostic protocols and the correlation of clinical states with inflammatory changes. According to current data, IL-6 can be a useful marker in the etiological diagnosis of acute gastroenteritis. Increased IL-6 values are correlated with the bacterial etiology of the disease, and IL-8 shows high values in this pathology, indicating its suitability as a biomarker. According to available data, both of the studied cytokines, IL-6 and IL-8, show increased values during episodes of acute gastroenteritis which can be correlated with the severity of the pathology. In conclusion, these cytokines could form the basis of an etiological, prognostic and diagnostic protocol through correlation with severity of inflammation which would be quick, cheap and easy to assess.

However, so far, both cytokines have rarely been studied in acute gastroenteritis in children or adults. Therefore, we believe that future studies, performed with larger cohorts, are necessary and warranted to establish an effective management protocol in acute diarrheal disease in the pediatric population.
